# Hemodynamic and antiemetic effects of prophylactic hyoscine butyl-bromide during cesarean section under spinal anesthesia: a randomized controlled trial

**DOI:** 10.1186/s12871-022-01659-9

**Published:** 2022-04-21

**Authors:** Mostafa Samy Abbas, Shimaa Abbas Hassan, Ahmed Mohamed Abbas, Amr Mohamed Thabet, Ahmed Mostafa Thabet, Magdy Mohammed Mahdy

**Affiliations:** 1 Anesthesia and Intensive Care department, Faculty of Medicine, Assiut University, Assiut, Egypt; 2 Obstetrics and Gynaecology department, Faculty of Medicine, Assiut University, Assiut, Egypt

**Keywords:** Bradycardia, Cesarean section, Hyoscine N-Butylbromide, Spinal anesthesia, Vomiting

## Abstract

**Background:**

Abrupt bradycardia and hemodynamic instability during spinal anesthesia for cesarean section are not uncommon and are considered as one of the primary causes of intraoperative nausea and vomiting (IONV). We hypothesized that prophylactic use of hyoscine butyl-bromide (HBB) could improve hemodynamics and reduce IONV in parturients undergoing cesarean section.

**Methods:**

A randomized, double-blind placebo-controlled trial was carried out in a tertiary university hospital, patients scheduled for elective cesarean section were equally randomized to receive either IV HBB 20 mg in 1 ml (Hyoscine group) or the same volume of 0.9% saline (Control group), one minute after spinal anesthesia. The primary endpoint was the incidence of intraoperative bradycardia (HR < 50 beats min^**−1**^). Secondary endpoints included changes in mean arterial blood pressure (MAP), the incidence of Intraoperative and Postoperative nausea or vomiting (IONV & PONV), the fetal heart rate and, Apgar score.

**Results:**

Of the 160 subjects randomized, 80 received HBB and 80 received placebo. There was a significant reduction in the incidence of the primary endpoint of intraoperative bradycardia (HR < 50 beats min^**−1**^) in the Hyoscine group (0% vs 10%; OR = 0.05, 95% CI = [0.003, 0.93]; *P* = 0.004) compared with placebo. MAP showed an insignificant difference between groups over time. HBB significantly decreased incidences of IONV and PONV (*p* = 0.002 & 0.004) respectively.

**Conclusions:**

In parturients undergoing cesarean section under spinal anesthesia, pretreatment with intravenous HBB was a safe measure for both the mother and the baby to reduce the risk of severe intraoperative bradycardia, but not hypotension. Furthermore, it was associated with less incidence of both IONV and PONV.

**Trial registration:**

https://clinicaltrials.gov/ct2/show/NCT04069078

## Background

In developing countries, anesthetic complications, particularly abrupt bradycardia progressing to sinus arrest, account for about 14% of maternal deaths amongst those undergoing Cesarean section (CS) [1]. Many of the reported causes of anesthesia-related deaths can be avoided with proper training and resources [2].

Furthermore, the unopposed vagal activity and maternal hypotension that occurs with sympathetic block associated with spinal anesthesia are one of the primary causes of intraoperative nausea and vomiting (IONV) [3, 4].

Unlike atropine, Hyoscine butyl-bromide (HBB) is an anticholinergic with a quaternary ammonium structure making it unable to cross the blood–brain barrier and has lower placental transfer, so it could be more favorable for use with pregnancy [5, 6]. Furthermore, the similar compound Scopolamine was described before to have antiemetic effects [7]. However, because of the undesirable side effects of scopolamine, its use as an antiemetic prophylaxis becomes questionable [8] and HBB could be a better alternative.

We hypothesized that prophylactic use of HBB could prevent post spinal bradycardia with better hemodynamics and less incidence of IONV in parturients undergoing spinal anesthesia for CS.

## Methods

### Ethics approval and consent to participate

The current study was a prospective single-center randomized, double-blind placebo-controlled trial registered at www.clinicaltrials.gov (NCT04069078) and first posted on August 28, 2019. The study was carried out in Assiut University Hospital, Egypt, between October 2019 and February 2020. This study was approved by the medical ethics committee of Assiut university with approval number [17300311]. All patients provided written informed consent. All methods were carried out in accordance with the Declaration of Helsinki 2013.

### Participants’ selection

All patients 18–40 years of age, American Society of Anesthesiologists (ASA) classification class I and II and scheduled for elective or semi-elective surgery (category 3 and 4 Cesarean section) under spinal anesthesia for a Single baby pregnancy of more than 32 weeks were included in this study. Exclusion criteria were Patient’s height < 150 or > 180 cm, Body mass index (BMI) > 35 kg m^**−2**^, Contraindication or refusal to undergo regional anesthesia, Baseline bradycardia (HR < 60 beats min^**−1**^), or any cardiovascular disease including arrhythmias, Patients on β-adrenergic blockers or any drugs that may alter the normal response to the study drug, Associated medical problem with pregnancy (as hypertension, diabetes mellitus, hepatic impairment or renal impairment).

### Blinding and randomization

Patients were allocated in 1: 1 ratio into the two study groups using a web-based randomizer (https://www.randomizer.org/) to generate codes placed within sealed, opaque, sequentially numbered envelopes by a research assistant who was not involved in patient care or assessment. This assistant also prepared the study solutions in identical syringes that were labeled ''study drug'' according to the assigned group as follow:

#### Hyoscine butyl bromide group (Group H)

Patients received IV study solution, which is hyoscine butyl-bromide 20 mg in 1 ml, one minute after spinal anesthesia.

#### The control group (Group C)

Patients received 1 ml of IV normal saline as a placebo one minute after spinal anesthesia.

Meanwhile, both patient and the anesthesiologists conducting anesthesia and assessing outcomes were blinded to patient allocation.

### Interventions

The preoperative anesthetic assessment was carried out including patients perinatal and general medical history, examination including baseline heart rate (HR), Blood pressure (BP) recording (three readings of HR and BP were averaged to give the baseline value for maternal HR and BP) and review of patients’ coagulation profile.

Fasting for 8 h before the operation was acceptable. They were also premedicated with oral sodium citrate (30 mL, 0.3 molar) one hour before the operation as anti-aspiration prophylaxis.

In the operating room in all patients, large-bore intravenous access (18 gauge) was inserted in the left dorsum of the hand, and they were preloaded with 15 mL/kg of Ringer's solution intravenously. The 1st 500 ml was transfused by a pressure bag.

All patients were connected to standard routine monitoring [non-invasive blood pressure (NIBP), electrocardiography, and peripheral oxygen saturation.

With the parturient in a sitting position, spinal anesthesia was conducted using the midline approach after proper antiseptic cleaning and draping in the lumbar vertebral interspace of L3-L4 or a level below using a 25-gauge Quincke needle. After obtaining free flow of cerebrospinal fluids, 3 ml intrathecal solution of hyperbaric bupivacaine 0.5% in a dose of 12.5 mg (2.5 ml) mixed with morphine in a dose of 200 µg (0.5 ml was withdrawn from a syringe containing 4 mg morphine sulfate diluted in 10 ml normal saline) were injected intrathecally.

All patients were then made to lie supine, and a wedge was placed below the right hip to give a left lateral tilt. Then the premade study syringe was given IV immediately. Block-level was tested every five minutes using a frozen plastic ampule of sterile water along the midclavicular line to check the response to cold, and the highest sensory level obtained was registered. Surgeons were allowed to proceed with surgery after attaining a block level of at least up to T6.

### Data collection

Sociodemographic patient's profile: age, sex, weight, height, and ASA physical status were recorded. HR and the mean arterial blood pressure (MAP) were recorded at 0 (baseline), 1, 3, 6, 9, 15,20, 25, 30, 40- and 50-min following administration of the study drugs respectively until the end of surgery. And then every two hours for 6 h postoperatively.

Bradycardia (HR < 50 beats min^**−1**^) was treated with intravenous atropine 0.5 mg. Clinically significant hypotension was defined as MAP < 60 mmHg or a decrease of MAP > 20% from the baseline, and if developed, was treated with iv ephedrine sulfate boluses of 6 mg as required. Amount of atropine and ephedrine required, sensory level achieved at 15 min of Spinal anesthesia, the incidence of intraoperative and postoperative nausea or vomiting (PONV), presence of intraoperative chest pain, and intraoperative and postoperative confusion were recorded till six hours postoperative.

Nausea and/or vomiting with stable hemodynamics were treated by iv injection of 4 mg ondansetron. Incidence of postoperative itching was monitored and treated with 25 mg iv diphenhydramine. Respiratory depression (Respiratory rate < 8 breaths/min or SPO2 < 90%) was managed with oxygen therapy, non-invasive or invasive ventilation as appropriate.

### Outcome measures

The primary outcome measure was the incidence of maternal bradycardia (HR < 50 beats min^**−1**^). The secondary outcome measures were the changes in MAP, the incidences of IONV and PONV and, the fetal heart rate and Apgar score at one and five minutes after delivery.

### Sample size

Data from a previous study reported 17% incidence of bradycardia within controls in parturients undergoing spinal anesthesia for CS [9]. Based on a power of 80% and a confidence level of 95%, the required sample size was calculated as 71 per group (OpenEpi, version 3, open-source calculator) to detect a bradycardia frequency of less than 3% in the Hyoscine-treated group. Considering of dropouts, 80 patients were recruited in each group.

### Statistical analysis

Data were collected and firstly checked for normality of distribution through the Kolmogorov–Smirnov test. Data are presented as mean (SD) or number and ratio. Groups' categorical data were compared through the Chi-square test. Continuous parametric data were compared by unpaired t-test, whereas nonparametric data by Mann Whitney U test (between groups). Changes over time in HR and MAP between and within the study groups and, comparing values at each time point, were analyzed by repeated-measures ANOVA followed by a post hoc Bonferroni test to identify significant differences. Haldane-Anscombe correction was used to calculate OR (odds ratio) when the frequency in one group equals "0". Data were investigated using the computer program IBM, SPSS (Statistical Package for Social Sciences), Version 22, 2015. The *P*-value < 0.05 was reflected statistical significance.

## Results

**Fig. 1:**
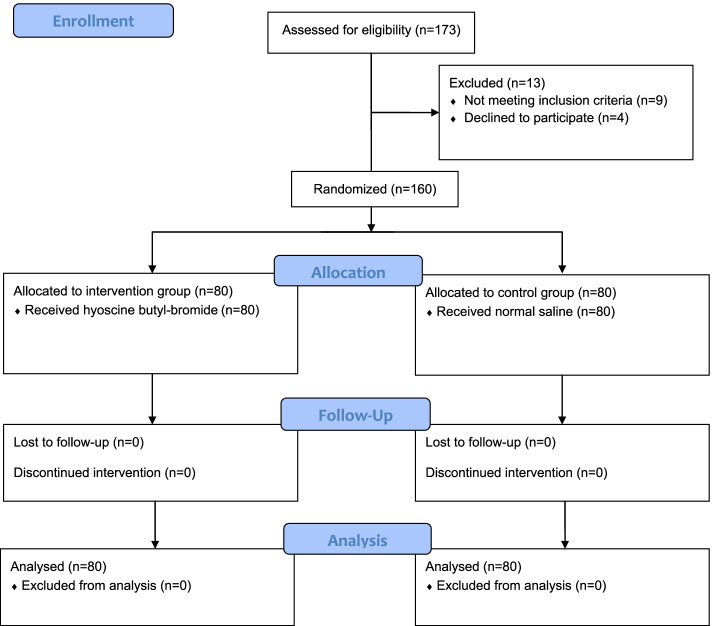
CONSORT flowchart showing patient recruitment

The recruitment of subjects is shown in Fig. 1. A total of 173 patients were screened for enrollment in this study. Thirteen patients were excluded (five had a body mass index > 35 kg m^**−2**^, two had preeclampsia, two received general anesthesia and, four declined to participate). One hundred sixty parturients completed the study with available data for the final analysis.

Patient characteristics, sensory block level, and operative time were comparable among groups (Table 1).

**Table 1:** Subjects’ characteristics, clinical data, and operative time

		Group C (*N* = 80)	Group H (*N* = 80)	*P* value
Age (years)		27.8 (4.5)	28.0 (4.7)	0.85
Weight (kg)		78.0 (16.9)	77.9 (15.3)	0.98
Height (cm)		166.1 (8.2)	167.4 (8.6)	0.31
BMI (kg m-2)		29.6 (24.2—32.9)	28.0 (23.3—32.9)	0.72
ASA N (%)	ASA 1	80 (100%)	80 (100%)	1
	ASA 2	0 (0)	0 (0)	NS
Sensory level at 15 min (dermatome)		T4 (T3—T4)	T4 (T2 -T 4)	0.21
Operative time (minutes)		30 (26—40)	30 (25—41)	0.62

Data are presented as mean (S.D.), median (25-75th percentiles) or number (%)

*BMI* Body mass index, *NS* Not significant, *ASA* American society of anesthesiologists

*P*-value < 0.05 was considered statistically significant

The incidence of maternal bradycardia (HR < 50 beats min^**−1**^) was calculated to be 0% for the Hyoscine group and 10% for the control group. The relative odds of bradycardia was 95% lower comparing subjects receiving hyoscine to control (OR = 0.05, 95% CI = [0.003, 0.93]; *P* = 0.004).

Moreover, when considering maternal bradycardia as HR < 60 beats min^**−1**^, the incidence was significantly much lower in the hyoscine group in comparison to the control group. (1.3% vs 18.8%; OR = 0.06, 95% CI = [0.007, 0.43]; *P* < 0.001). (Table 2).

**Table 2:** Intraoperative and postoperative side effects and interventions

Variables	Group C (*N* = 80)	Group H (*N* = 80)	*P* value
Bradycardia (HR < 50) N (%)	8 (10)	0 (0)	0.004
Bradycardia (HR < 60) N (%)	15 (18.8)	1 (1.3)	< 0.001
IONV incidence N (%)	28 (35%)	11 (13.8%)	0.002
PONV incidence N (%)	41 (51.3%)	23 (28.8%)	0.004
Patients required Atropine N (%)	11 (13.8%)	0 (0%)	0.001
Patients required Ephedrine N (%)	61 (76.3%)	38 (47.5%)	< 0.001
Total Atropine dose (mg)	0 (0—0)	0 (0—0)	0.001
Total Ephedrine dose (mg)	6 (6—18)	0 (0—6)	< 0.001
Intravenous Fluid volume given (ml)	1000 (1000—1500)	1000 (1000—1437.5)	0.58
Intraoperative Chest Pain N (%)	0 (0%)	2 (2.5%)	0.16
Intra. & Postoperative Confusion N (%)	0 (0%)	0 (0%)	NS

Data are presented as median (25-75th percentiles) or number (%)

*IONV* Intra-Operative Nausea and Vomiting, *PONV* Post-Operative Nausea and Vomiting, *NS* Not significant

*P*-value < 0.05 was considered statistically significant

**Fig. 2:**
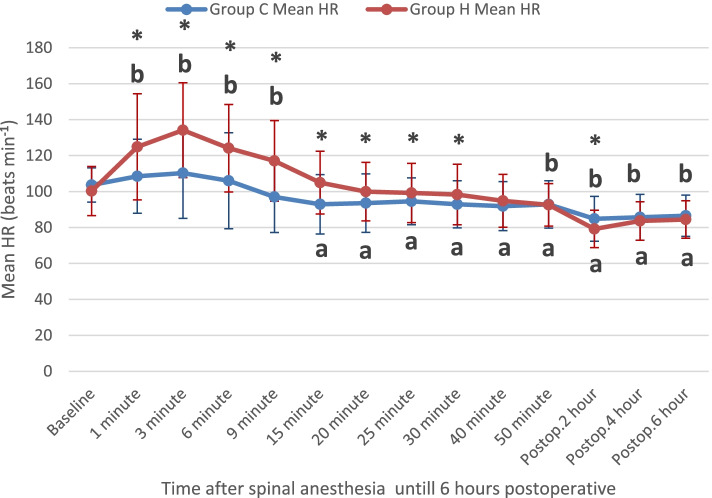
Serial changes in mean heart rate in both groups over time calculated using repeated-measures ANOVA followed by a post hoc Bonferroni test to identify significant differences. Data are shown as mean (standard deviation, S.D.). HR: heart rate. Group H: hyoscine group. Group C: control group. Postop: postoperative. (*), *P*-value < 0.05 comparing both groups at the same time point. **a** *P* < 0.05 compared to the baseline value in group C **b** *P* < 0.05 compared to the baseline value in group H. *P* < 0.05 was considered statistically significant

Serial changes in heart rate are shown in Fig. 2. Analysis of data showed that heart rate changes over time were significantly different between subjects in both groups [F(1,158) = 20.8, *P* < 0.001], with the peak heart rate in the hyoscine group at 3 min after injection then came to baseline within 20 min.

**Fig. 3:**
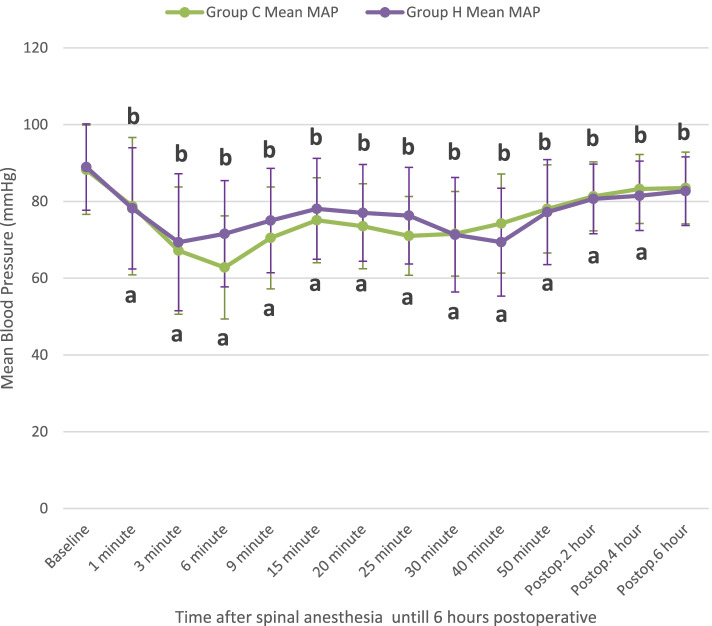
Serial changes in mean arterial pressure (mmHg) in both groups over time calculated using repeated-measures ANOVA. Data are shown as mean (standard deviation, S.D.). MAP: mean arterial pressure. Group H: hyoscine group. Group C: control group. Postop: postoperative. **a** *P* < 0.05 compared to the baseline value in group C. **b** *P* < 0.05 compared to the baseline value in group H. *P* < 0.05 was considered statistically significant

Changes in MAP over time are shown in Fig. 3. MAP showed an insignificant difference between groups over time [F (1,158) = 1.87, *P* = 0.17].

Results showed a significantly lower incidences of IONV and PONV in the hyoscine group compared to the control group (13.8% vs 35%; OR = 0.30, 95% CI = [0.14, 0.65]; *P* = 0.002) and, (28.8% vs 51.3%; OR = 0.38, 95% CI = [0.20, 0.74]; *P* = 0.004) respectively. (Table 2).

There were no significant differences in the incidences of Intraoperative Chest pain, intraoperative and postoperative confusion between the study groups (Table 2). Neonatal outcomes were similar between groups (Table 3). Patients required ephedrine or atropine, and the doses used are summarized in Table 4.

**Table 3:** Neonatal outcomes

Variables	Group C (*N* = 80)	Group H (*N* = 80)	*P* value
Neonatal HR 1 min (bpm)	133.8 (20.1)	134.9 (18.5)	0.70
Neonatal HR 5 min (bpm)	152.3 (14.2)	153.2 (13.9)	0.69
APGAR Score 1 min	8 (7—8.3)	8 (7—9)	0.66
APGAR Score 5 min	9 (9—10)	10 (9—10)	0.77

Data are presented as mean (S.D.) or median (25-75th percentiles)

*HR* Heart Rate

*P*-value < 0.05 was considered statistically significant

**Table 4:** Frequencies of patients required atropine and ephedrine in each group

Groups	**Atropine dose (mg)**			**Ephedrine dose (mg)**						
	0	0.5	1	0	6	12	18	20	24	30
Group C (*N* = 80)	69 (86.25%)	9 (11.25%)	2 (2.5%)	19 (23.75%)	23 (28.75%)	17 (21.25%)	13 (16.25%)	3 (3.75%)	4 (5%)	1 (1.25%)
Group H (*N* = 80)	80 (100%)	0 (0%)	0 (0%)	42 (52.5%)	26 (32.5%)	9 (11.25%)	2 (2.25%)	0 (0%)	1 (1.25%)	0 (0%)

Data are presented as number (%)

## Discussion

The present study shows that prophylactic use of I.V. HBB 20 mg with spinal anesthesia prevents intraoperative bradycardia during CS compared with placebo.

The blockade of the preganglionic cardio accelerator fibers originating between T1 to T4 may progress rapidly to complete heart block or asystole, which necessitates intense monitoring and rapid intervention [10]. Furthermore, the reflexes initiated due to hypotension and the decreased preload following spinal anesthesia, might cause sever bradycardia [11].

Despite using ephedrine which was found in a meta-analysis of vasopressor drugs used in CS [12] to be associated with the least incidence of maternal bradycardia, the incidence of bradycardia within controls in our study was found to be 10% (HR < 50 beats min^**−1**^).

To our knowledge, few studies investigated bradycardia as a target primary outcome and examined pharmacological interventions to decrease its incidence. Our results are parallel with a previous report by Chamchad D and others, who stated that prophylactic glycopyrrolate administered before spinal anesthesia prevents bradycardia during Cesarean section (*p* = 0.0248). They found the incidence of bradycardia to be 17% between controls but, they defined bradycardia as HR < 60 beats min^**−1**^ [9].

In contrast to our results, Tarkkila and Kaukinen, found insignificant effect of prophylactic glycopyrrolateon the incidence of bradycardia in an observational cohort of 1,881 patients. However, their study was without randomization or blinding and did not specify the number of patients who received glycopyrrolate or doses given [13]. Also, Kee and co-workers showed no significant effect on the incidence of bradycardia when using glycopyrrolate pretreatment before phenylephrine infusion during spinal anesthesia for CS (*p* = 0.50). Unlike our study, kee and co-authors calculated the sample size for cardiac output as a primary outcome [14].

The incidence of bradycardia in parturients undergoing spinal anesthesia varied between studies to be as low as 4.2% & 10% in some studies [14, 15] and as high as 46% in other studies [16] according to the definition of bradycardia and the vasopressor used.

Atropine is perhaps the most commonly used anticholinergic drug. However, Hyoscine butyl bromide is a semisynthetic derivative of hyoscine hydrobromide (scopolamine), which has a quaternary ammonium structure that results in lower transfer through the blood–brain barrier causing less incidence of confusion and other central side effects [5, 6]. our results showed no difference in the incidence of intraoperative and postoperative confusion between the study groups.

While the vagal blockade of glycopyrrolate persists for two to three hours [17], our study found the peak heart rate in the hyoscine group to be at 3 min after injection and came to baseline within 20 min. This makes HBB more preferable than glycopyrrolate to avoid long periods of tachycardia. Regarding the effect on blood pressure, HBB did not show any significant difference in MAP between groups.

Our results showed that HBB reduced the incidence of intraoperative nausea and vomiting significantly. These results were significant in our study because we used ephedrine, which carries a higher incidence of nausea and vomiting than the other vasopressor agents [12].

The exact etiology of intraoperative nausea during spinal anesthesia is unknown. However, it is suggested that the unopposed vagal activity that occurs with sympathetic block during spinal anesthesia is the cause. Supporting this theory is the observation that atropine was more effective in relieving nausea during spinal anesthesia than was the elevation of the blood pressure with vasopressors [18].

Intrathecal opioids are associated with a 30%–70% incidence of postoperative nausea and vomiting [19, 20]. In our study, prophylactic HBB was associated with a significant reduction in the incidence of nausea and vomiting in the first six postoperative hours. Parallel to our results, Harnett and others compared the antiemetic efficacy of transdermal scopolamine, IV ondansetron, and placebo during the first postoperative day. They found scopolamine to significantly reduce the incidence of nausea and vomiting when compared to the placebo group in parturients receiving intrathecal morphine during cesarean section [7]. However, concerns have been raised regarding the use of scopolamine as antiemetic prophylaxis because of its short duration of action and the undesirable side effects (e.g., visual disturbances, dry mouth, dizziness, agitation, drowsiness) resulting from large peak plasma concentrations [8].

Despite being a quaternary ammonium compound, HBB can target the chemoreceptor trigger zone due to the absence of a well-developed blood–brain barrier in the medulla oblongata. This central effect potentiates the antiemetic effects that it produces through local action on the smooth muscles of the gastrointestinal tract [21].

Regarding neonatal outcome and in accordance with many studies [22–26], our results showed no significant effect of HBB on APGAR score at 1 & 5 min after birth. In a meta-analysis done by Zaynab and colleagues to assess the effectiveness of hyoscine n-butyl bromide in labor progress, analysis of 11 studies involving 1682 participants showed an insignificant difference in the APGAR score at 1 min between HBB and controls (mean difference =—0.04; 95% CI: [− 0.09, 0.01]). Also, the results of seven studies (involving 1453 neonates) showed an insignificant effect of HBB on APGAR score at 5 min after birth (mean difference =  − 0.03; 95% CI: [− 0.08, 0.03]) [27].

This study is not without limitations; first, we used ephedrine as a vasopressor which appears to be the most likely agent to affect fetal and maternal outcomes adversely, but norepinephrine and phenylephrine are not available in limited-resource areas, in addition to carrying the risk of drug errors in wards outside of Critical Care Units [12]. So that we used ephedrine because of its availability in our community, the ease of its use and, because it has the least effect on the incidence of bradycardia [12]. Second, our results showed an insignificant difference between groups in Side effects like confusion and chest pain, but we did not monitor for dry mouth, constipation, urinary retention, reflux and flushing. However, by data analysis from five studies for the effect of HBB on mouth dryness in a previous metanalysis [27] the difference between HBB and controls was insignificant.

## Conclusions

pretreatment with intravenous HBB 20 mg in parturients undergoing CS under spinal anesthesia is a safe measure for both the mother and the baby to prevent the risk of severe intraoperative bradycardia with no noticeable effect on maternal hypotension. Moreover, it was associated with less incidence of both intraoperative and postoperative nausea and vomiting. Future studies are required to confirm our results, especially the effect on nausea and vomiting as a significant complication of hypotension and intrathecal morphine and as a measure of the quality of recovery after cesarean section.

## Data Availability

The datasets used and/or analyzed during the current study are available from the corresponding author on reasonable request.

## Abbreviations

CS: Cesarean section
IONV: Intraoperative nausea and vomiting
HBB: Hyoscine butyl-bromide
ASA: American Society of Anesthesiologists
HR: Heart rate
BP: Blood pressure
NIBP: Non-invasive blood pressure
MAP: Mean arterial blood pressure
PONV: Postoperative nausea and vomiting
SD: Standard deviation
OR: Odds ratio
SPSS: Statistical Package for Social Sciences
CI: Confidence interval

## References

[1] Sobhy S, Zamora J, Dharmarajah K, Arroyo-Manzano D, Wilson M, Navaratnarajah R (2016). Anaesthesia-related maternal mortality in low-income and middle-income countries: a systematic review and meta-analysis. Lancet Glob Health.

[2] White MC, Rakotoarisoa T, Cox NH, Close KL, Kotze J, Watrous A (2019). A mixed-method design evaluation of the SAFE obstetric anaesthesia course at 4 and 12–18 months after training in the Republic of Congo and Madagascar. Anesth Analg.

[3] Datta S, Alper MH, Ohtheimer GW, Weiss JB (1982). Method of ephedrine administration and nausea and hypotension during spinal anesthesia for cesarean section. Anesthesiology.

[4] Racké K, Schwörer H (1991). Regulation of serotonin release from the intestinal mucosa. Pharmacol Res.

[5] Kivalo I, Saarikoski S (1977). Placental transmission of atropine at full-term pregnancy. Br J Anaesth.

[6] Ali-Melkkilä T, Kaila T, Kanto J, Iisalo E (1990). Pharmacokinetics of glycopyrronium in parturients. Anaesthesia.

[7] Harnett MJ, O’Rourke N, Walsh M, Carabuena JM, Segal S (2007). Transdermal scopolamine for prevention of intrathecal morphine-induced nausea and vomiting after cesarean delivery. Anesth Analg.

[8] Kranke P, Morin AM, Roewer N, Wulf H, Eberhart LH (2002). The efficacy and safety of transdermal scopolamine for the prevention of postoperative nausea and vomiting: a quantitative systematic review. Anesth Analg.

[9] Chamchad D, Horrow JC, Nakhamchik L, Sauter J, Roberts N, Aronzon B (2011). Prophylactic glycopyrrolate prevents bradycardia after spinal anesthesia for Cesarean section: a randomized, double-blinded, placebo-controlled prospective trial with heart rate variability correlation. J Clin Anesth.

[10] Cotoia A, Mirabella L, Raimondo P, Cinnella G, Malvasi A, Tinelli A, Di Renzo GC (2017). Complications of Regional Anesthesia. Management and Therapy of Late Pregnancy Complications: Third Trimester and Puerperium.

[11] Mercier F, Bonnet M-P, la Dorie De A, Moufouki M, Banu F, Hanaf A (2007). Spinal anaesthesia for caesarean section: fluid loading, vasopressors and hypotension. Ann Fr Anesth Reanim.

[12] Singh PM, Singh NP, Reschke M, Kee WDN, Palanisamy A, Monks DT (2020). Vasopressor drugs for the prevention and treatment of hypotension during neuraxial anaesthesia for Caesarean delivery: a Bayesian network meta-analysis of fetal and maternal outcomes. Br J Anaesth.

[13] Tarkkila PJ, Kaukinen S (1991). Complications during spinal anesthesia: a prospective study. Reg Anesth Pain Med.

[14] Kee WN, Lee S, Khaw K, Ng F (2013). Haemodynamic effects of glycopyrrolate pre-treatment before phenylephrine infusion during spinal anaesthesia for caesarean delivery. Int J Obstet Anesth.

[15] Fu F, Xiao F, Chen W, Yang M, Zhou Y, Kee WDN (2020). A randomised double-blind dose–response study of weight-adjusted infusions of norepinephrine for preventing hypotension during combined spinal–epidural anaesthesia for Caesarean delivery. Br J Anaesth.

[16] McDonnell N, Paech M, Muchatuta N, Hillyard S, Nathan E (2017). A randomised double-blind trial of phenylephrine and metaraminol infusions for prevention of hypotension during spinal and combined spinal–epidural anaesthesia for elective caesarean section. Anaesthesia.

[17] Ali-Melkkilä T, Kaila T, Kanto J (1989). Glycopyrrolate: pharmacokinetics and some pharmacodynamic findings. Acta Anaesthesiol Scand.

[18] Ward RJ, Kennedy WF, Bonica JJ, Martin WE, Tolas AG, Akamatsu T (1966). Experimental evaluation of atropine and vasopressors for the treatment of hypotension of high subarachnoid anesthesia. Anesth Analg.

[19] Dahl JB, Jeppesen IS, Jørgensen H, Wetterslev J, Møiniche S (1999). Intraoperative and postoperative analgesic efficacy and adverse effects of intrathecal opioids in patients undergoing cesarean section with spinal anesthesia a qualitative and quantitative systematic review of randomized controlled trials. Anesthesiology.

[20] Nortcliffe SA, Shah J, Buggy D (2003). Prevention of postoperative nausea and vomiting after spinal morphine for Caesarean section: comparison of cyclizine, dexamethasone and placebo. Br J Anaesth.

[21] Glare P, Miller J, Nikolova T, Tickoo R (2011). Treating nausea and vomiting in palliative care: a review. Clin Interv Aging.

[22] Edessy M, EL-Darwish A, Nasr A, Ali A, El-Katatny H, Tammam M (2015). Different modalities in first stage enhancement of labor. Gen Health Med Sci.

[23] Barau DD, Agida ET, Onafowokan O, Adebayo FO (2018). Effect of Hyoscine Butyl Bromide on the Course of Labour. Open J Obstet Gynecol.

[24] Treviño-Salinas EM, del Campo GC-M, Ayuzo-del Valle C, Guzmán-López A, Soria-López JA, Iglesias-Benavides JL (2015). Effect of hyoscine butylbromide on cervical dilation during labor. Medicina universitaria.

[25] Babapour N, Hoseini M, Zarif NP (2021). Rectal versus Intramuscular Hyoscine: its Effects on Shortening the First Stage of Labor in Term Primigravid Women. Int J Pediatr.

[26] Maged AM, Sorour EH, ElSadek MM, Hassan SM, Shoab AY (2021). A randomized controlled study of the effect of hyoscine butylbromide on duration of labor in primigravida women with prolonged labor. Arch Gynecol Obstet.

[27] Mohaghegh Z, Abedi P, Faal S, Jahanfar S, Surdock A, Sharifipour F (2020). The effect of hyoscine n-butylbromide on labor progress: a systematic review. BMC Pregnancy Childbirth.

